# The molecular and immune landscape of the forkhead-box gene family in different subtypes of breast cancer

**DOI:** 10.1016/j.gendis.2025.101911

**Published:** 2025-10-29

**Authors:** Xiaoman Bi, Liyang Chen, Deng Wu, Dahua Xu, Dehua Zheng, Zhizhou Xu, Zhenling Wan, Shaoping Zheng, Kongning Li, Shaojiang Zheng

**Affiliations:** a Key Laboratory of Emergency and Trauma of Ministry of Education, Engineering Research Center for Hainan Biological Sample Resources of Major Diseases, School of Intelligent Medicine and Technology, Big Data Research Center, The Hainan Branch of National Clinical Research Center for Cancer, The First Affiliated Hospital, Hainan Medical University, Haikou, Hainan 571199, China; b Key Laboratory of Environmental Medical Engineering and Education Ministry, School of Public Health, Southeast University, Nanjing, Jiangsu 210009, China; c Department of Pathology, Hainan Women and Children's Medical Center, Hainan Medical University, Haikou, Hainan 570206, China; d Department of Ultrasound, Union Hospital, Tongji Medical College, Huazhong University of Science and Technology, Wuhan, Hubei 430022, China; e Hainan Engineering Research Center for Health Big Data, Hainan Medical University, Haikou, Hainan 570102, China

Breast cancer is the most common cancer in women, and is inherently heterogeneous at both the morphological and molecular levels.^1^ The evolutionarily conserved forkhead box (FOX) transcription factor family plays an essential role in various biological processes. Although the involvement of several FOX genes in breast cancer has been reported,^2^ limited work has been performed on the FOX gene family profiling in different subtypes of breast cancer, especially regarding the cell type-specific variation of the FOX gene family members.

In this study, we examined somatic mutations and copy number variations of 49 FOX genes among five PAM50 intrinsic breast cancer subtypes from The Cancer Genome Atlas (TCGA) cohort. Around 10% (91 out of 986) of patients with breast cancer carried somatic mutations in the FOX genes (Fig. S1A). FOXA1 has the highest mutation rate, occupying more than 20% of patients with FOX gene mutations regardless of the LumA, LumB, and HER2E subtypes (Fig. S1B). Missense mutations are the most common type of FOXA1 gene mutation, and they frequently occur in the FH domain (Fig. S1C). In contrast to the low prevalence of somatic mutation, FOX genes show significantly altered copy number variations in breast tumor patients (Fig. S1D).

To investigate the gene expression pattern of the FOX gene family in breast cancer, differential gene expression analysis was performed between 1084 breast cancer patient samples and 113 normal samples from TCGA. A total of 35 FOX genes showed significant differences between breast cancer patients and normal samples (Fig. 1A; Table S1). In consistent with previous studies,^3^ FOXM1 was found to be an oncogenic transcription factor and was significantly overexpressed (Fig. 1A; Fig. S2A). In addition, other FOX genes, including FOXN3 and FOXO4, also showed significant changes (Fig. 1A). We examined these findings in three additional independent datasets, and consistent results were observed (Fig. S2B). To investigate whether the diverse regulation of FOX genes was determined by individual heterogeneity, a PAM50 subtype-dependent differential expression gene analysis was performed. As expected, the most significantly altered FOX genes, like FOXM1 and FOXO1, were consistently up- and down-regulated across all five subtypes (Fig. 1A). FOXA1 and FOXN2 were specifically down-regulated in the basal-like subtype and up-regulated in the other subtypes, whereas FOXC1 and FOXC2 exhibited the exact opposite pattern, which was specifically up-regulated in the basal-like group (Fig. 1B).

**Figure 1:**
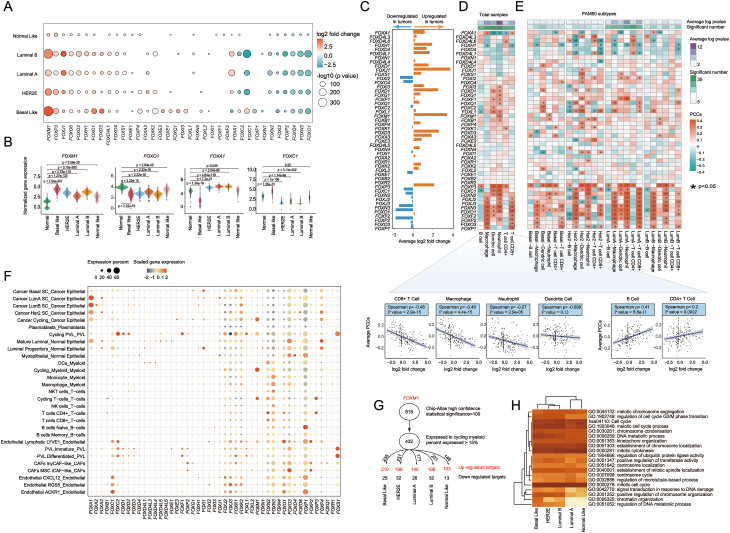
The multi-omics features of the FOX gene family in different subtypes of breast cancer. **(A)** Bubble plot of the differentially expressed FOX genes in various PAM50 subtypes. The size of bubbles represents the significance of the *P*-value. The red color represents the FOX genes up-regulated in tumor patients, and the blue represents the FOX genes down-regulated in tumors. **(B)** Violin plot of the normalized expression levels of the target FOX genes across all breast subtypes. **(C)** Average expression pattern of FOX genes between breast tumors and normal samples. **(D)** The correlation between FOX gene expression and immune cell infiltration in breast cancer. A dot plot illustrates the positive and negative relationship between FOX genes and each kind of cell type. **(E)** The correlation between FOX gene expression and immune cell infiltration in breast PAM50 subtypes. **(F)** Expression of the FOX genes in each kind of cell type. The expression of the whole list of FOX genes across the cell types is shown in the left dot plot. The dot size represents the expression percentage of the target FOX genes in each cell type, and the color represents the scaled gene expression. Target FOX gene expression is shown in the right t-SNE plots. **(G)** The FOXM1 target genes were collected from the Chip-Atlas, and then were filtered according to the percentage of expression in the FOXM1 highly expressed cell types, such as Cancer Basal SC_Cancer epithelial (Basal SC), Cancer LumA SC_Cancer epithelial (LumA SC), Cancer LumB SC_Cancer epithelial (LumB SC), and Cancer Her2 SC_Cancer epithelial (Her2 SC). The significantly differently expressed target genes of FOXM1 in each cell type were defined by their variation between tumor and normal samples. **(H)** Functional enrichment analysis of the cell type-specific target genes of FOXM1.

Accumulating evidence shows that FOX genes are involved in tumor immune infiltration.^4^ To investigate the mediating role of the FOX gene family in breast cancer, we calculated the correlation between the expression of FOX genes and the immune cell infiltration level. In general, a substantial positive correlation was observed, especially for the genes including FOXN3, FOXN2, FOXO1, FOXP2, and FOXC1, which were down-regulated in breast tumors (Fig. 1C). In contrast, the up-regulated genes, such as FOXA1 and FOXH1, displayed a negative correlation with the infiltration of immune cells. These findings suggest that both up- and down-regulated FOX genes result in a deactivated immunological environment, which was further verified by the fact that FOXP3, a regulatory T cell marker, significantly increased in tumors and simultaneously demonstrated a positive correlation with the infiltration immune cells among the tumor patients (Fig. 1C). The expression of FOX genes was significantly negatively correlated with the infiltrating immune cell types, including CD8 T cells and myeloid cells, such as macrophages, neutrophils, and dendritic cells (Fig. 1D; Table S2). Conversely, there was a positive correlation between the expression of FOX genes and B cells/CD4 T cells. Taking PAM50 subtypes into account, we were surprised to see that four PAM50 subtypes showed a similar pattern to the bulk samples, driven by a strong negative correlation between the FOX gene expression and the immune cell ratio (Fig. S3). These findings suggest that regardless of the tumor subtype, the variations of FOX genes were related to a deactivation of the immune response, which was mediated by decreased infiltration of myeloid cells.

To validate the hypothesis that different roles FOX genes play in carcinogenesis may be determined by the cell type-specific expression of these genes in breast tissues, a single-cell RNA transcriptome sequencing dataset, which contains 11 ER^+^ (Luminal A and Luminal B), 5 HER2^+^, and 10 triple-negative breast cancers from 26 breast tumor patients, was re-analyzed. A total of 29 cell types were identified and annotated using canonical lineage markers (Fig. S4A and S4B). FOXA1 is specifically highly expressed in the epithelial cells of Luminal A, Luminal B, and HER2E subtypes, and expressed in limited epithelial cells of the basal-like group (Fig. S4C; Table S3), which is consistent with the expression pattern between the PAM50 subtypes of breast cancers. Given the highest ratio of FOXA1 mutations found in breast tumors and the expression specific to epithelial cell-specific pattern, we suspect that FOXA1 mutations initiate breast tumorigenesis. However, FOXM1 mutation is only overexpressed in circulating immune cells (Fig. 1F), which challenges the conventional conclusion that FOXM1 is directly involved in the growth and proliferation of tumor cells.^5^ The activation of cycling immune cells in breast tumors, as demonstrated by the subtype-independent high expression of FOXM1, may be associated with the advancement of carcinogenesis. Simultaneously, FOXP3, another gene highly expressed in breast tumors, was exclusively overexpressed in CD4^+^ T and cycling T cells (Fig. 1F). The most down-regulated genes, FOXO1, FOXN3, and FOXP2, were highly expressed in non-tumor cells, indicating that tumor patients may have an inhibition of the normal cellular function. Additionally, FOXC1, a marker gene that is specifically highly expressed in endothelial cells, was specifically increased in the basal-like group. It may be related to the aggressive behavior and the enhanced vascular proliferation of the basal-like subtype. Taken together, single-cell transcriptomics reveal that FOX genes may target specific cell types and play roles in carcinogenesis.

To further employ the regulation of FOX genes on breast tumors, the target genes that co-express in the same cell type were evaluated. We gathered the high-confidence FOX-target interacted pairs from the Chip-Atlas database and retained the FOX-target pairs that were expressed in more than 15 % of the target cells for the following analysis. As the most up-regulated gene in breast cancers, FOXM1 is specifically highly expressed in the cycling myeloid cells. Accordingly, a total of 432 target genes from 818 high-confidence ones were detected in the cycling myeloid cells (Fig. 1G; Table S4). As expected, the majority of the target genes were significantly increased in breast tumors, which is highly consistent with the global up-regulation of FOXM1 in breast cancer. Functional enrichment analysis confirmed that the target genes of FOXM1 were directly involved in the mitotic cell cycle-associated functions and revealed an activated proliferation of cycling myeloid cells in breast cancer (Fig. 1H). We also conducted this target gene analysis on FOXA1 and FOXO1 (Fig. S5 and S6; Table S5 and S6).

In conclusion, our study combined single-cell transcriptome, bulk transcriptome, and genomic data of five different molecular subtypes of breast cancer to investigate FOX genes in breast cancer and offer a refreshing perspective for the multifaceted regulation of the FOX genes. This study provides a reasonable explanation for the heterogeneity of FOX genes in tumorigenesis based on their specific target cell types, contributing to a better understanding of the role FOX genes play in breast tumorigenesis.

## CRediT authorship contribution statement

**Xiaoman Bi:** Writing – review & editing, Supervision, Funding acquisition, Data curation, Writing – original draft, Investigation, Formal analysis, Conceptualization. **Liyang Chen:** Writing – original draft, Methodology, Formal analysis, Writing – review & editing, Software, Investigation, Data curation. **Deng Wu:** Writing – original draft, Methodology, Conceptualization, Writing – review & editing, Supervision, Formal analysis. **Dahua Xu:** Methodology, Data curation, Software, Formal analysis. **Dehua Zheng:** Methodology, Data curation, Software, Formal analysis. **Zhizhou Xu:** Methodology, Data curation, Software, Formal analysis. **Zhenling Wan:** Investigation, Resources. **Shaoping Zheng:** Writing – review & editing, Conceptualization, Supervision. **Kongning Li:** Supervision, Funding acquisition, Writing – review & editing, Project administration, Conceptualization. **Shaojiang Zheng:** Supervision, Project administration, Conceptualization, Writing – review & editing, Resources, Funding acquisition.

## Funding

This work was supported by the Hainan Provincial Natural Science Foundation (China) (No. 822QN462, 823RC581, 822MS171), the Program of Hainan Association for Science and Technology Plans to Youth R & D Innovation (China) (No. QCQTXM202212), Major Science and Technology Program of Hainan Province, China (No. ZDKJ2021040, ZDKJ2021038), the National Natural Science Foundation of China (No. 32160152, 81960528, 32400543), the specific research fund of The Innovation Platform for Academicians of Hainan Province, China (No. YSPTZX202208), Hainan Province Clinical Medical Center (China) (No. 2022341), and Academic Enhancement Support Program of Hainan Medical University (China) (No. XSTS2025139).

## Conflict of interests

The authors declared no conflict of interests.

## References

[1] Cancer Genome Atlas Network (2012). Comprehensive molecular portraits of human breast tumours. Nature.

[2] Wu S.Z., Al-Eryani G., Roden D.L. (2021). A single-cell and spatially resolved atlas of human breast cancers. Nat Genet.

[3] Cheng Y., Sun F., Thornton K. (2022). FOXM1 regulates glycolysis and energy production in multiple myeloma. Oncogene.

[4] Yi J., Tan S., Zeng Y. (2022). Comprehensive analysis of prognostic and immune infiltrates for FOXPs transcription factors in human breast cancer. Sci Rep.

[5] Zhang Y.L., Ma Y., Zeng Y.Q. (2021). A narrative review of research progress on FoxM1 in breast cancer carcinogenesis and therapeutics. Ann Transl Med.

