# Supplementary material for: The molecular and immune landscape of the forkhead-box gene family in different subtypes of breast cancer
Source: Genes Dis. 2025 Oct 29;13(3):101911. doi: 10.1016/j.gendis.2025.101911 (PMC12824906; doi:10.1016/j.gendis.2025.101911)
Supplement: Multimedia component 1 [file mmc1.docx]

**Supplementary Materials**

**Materials and Methods**

**Differential gene expression analysis**

Genomics and transcriptomics data of 1,098 breast cancer patient samples and 113 normal samples were downloaded from The Cancer Genome Atlas Program (TCGA). Seven samples of metastatic tumors were removed in the following analysis. PAM50 subgroups including 139 basal like samples, 67 HER2E samples, 420 Luminal A samples, 190 Luminal B samples and 23 Normal like samples were obtained from the clinical phenotypes. In total of 258 samples were not classified as any subtypes. The maftools was used to visualized and analyzed the FOX gene mutation across the samples. The DESeq2 R package was used for differential expression analysis, and genes with p<0.05 and |FC|>1.5 were considered as significant differential genes. An average expression was used if multiple samples were sequenced for one individual.

Breast tumor-associated transcriptomic datasets including GSE31448 (353 cancer samples, 4 normal samples), GSE65194 (167 cancer samples, 11 normal samples) and GSE42568 (104 cancer cases and 17 normal samples) were collected and validated the expression of FOX genes between tumor and normal ones. The limma package was used to obtain the significantly differentially expressed genes between tumor and normal samples. Genes with |FC| > 1.5 and p < 0.05 were defined as significantly changed genes and were analyzed in the following analysis.

**Immunoinfiltration analysis of the FOX gene family in subtypes of breast cancer**

TIMER2.0 database was use to retrieve data on the immune infiltration levels of 49 FOX gene family members in breast cancer and the correspondent six PAM50 subtypes (TIMER platform). The correlation between FOX genes and immune ratios were estimated by spearman correlation coefficient, the pairs with p<0.05 and |ρ|>0.2 were considered significant correlated.

**Single cell data analysis of the FOX gene family in breast cancer**

Single cell sequencing of 26 patients including 11 ER+, 5 HER2+ and 10 TNBCs (GSE176078) were downloaded from the Gene Expression Omnibus (GEO) database. Seurat package was used to perform the data normalization, scale, dimensionality reduction and clustering. In total of 29 different cell types were identified by integrated the cell type information as well as their specific markers. The DotPlot function was used to visualize and obtain the percent expression and average expression of 49 FOX genes in 29 types of cells.

**Enrichment analysis of key FOX target genes and FOX-related genes**

FOX target genes were obtained from Chip Atlas database with confidence score larger than 100. To estimate the cell type specific regulation of FOX genes, only the target genes expressed in more than 15% cells within the FOX highly expressed cell types were retained. And then, the differentially expressed target genes were obtained by overlapping to the PAM50 subgroups specific DEGs. The functional enrichment analysis of significantly changed target genes was analyzed by Metascape database with default settings, the terms with p value smaller than 0.05 were considered as significantly over-represented.

**Supplementary Figures:**


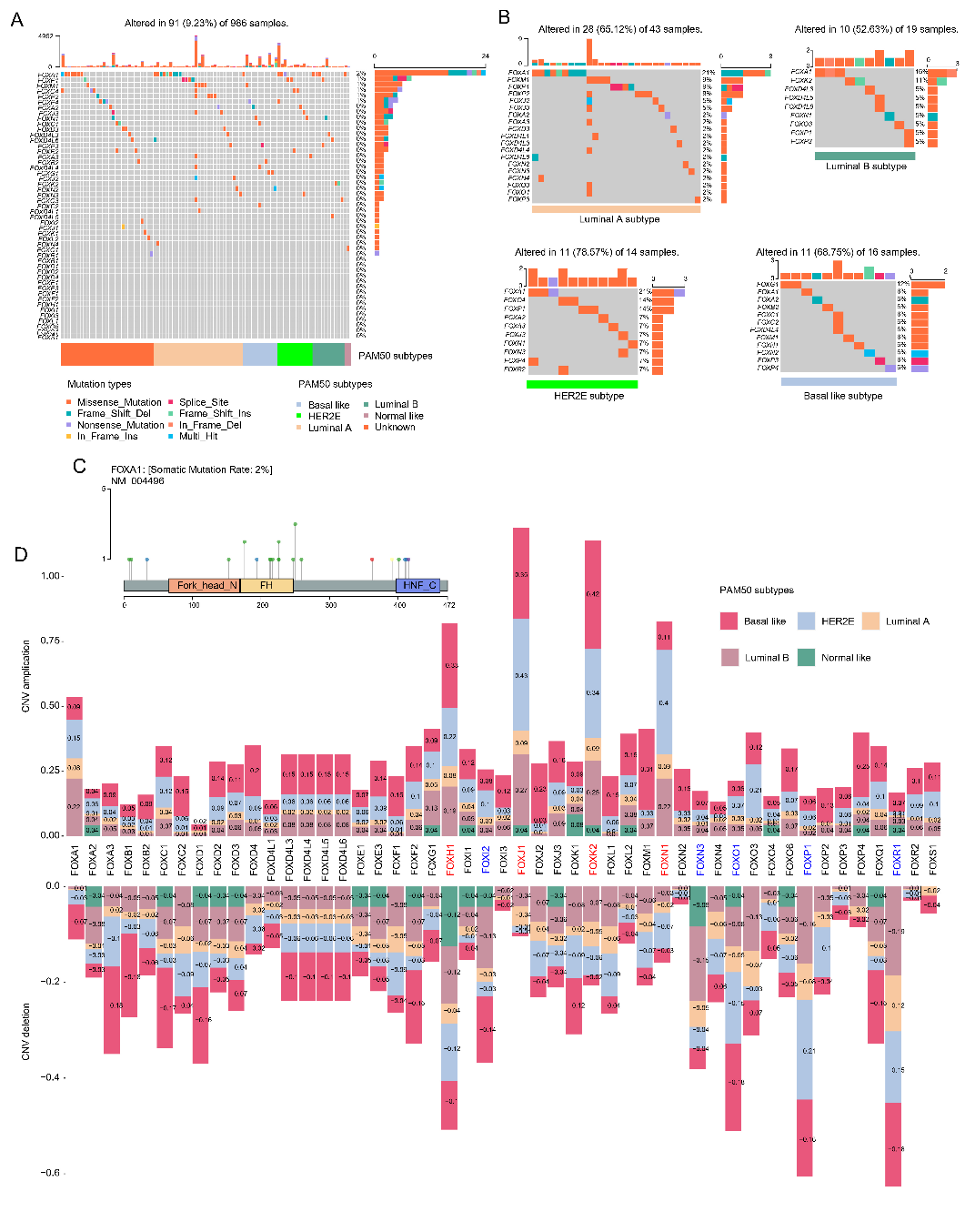


**Figure S1. Genomic variation of FOX family in breast cancers.** Waterfall plot representation of the mutational patterns of FOX family across the breast cancers (A) and the PAM50 subtypes (B), respectively. In each panel, the upper shows the mutation counts for each sample, and the left lists the FOX genes, which were ordered by their mutation ratio in breast cancers in the right side. (C) Mutation of FOXA1 in breast tumor patients. The Pfam protein domains and the mutation location are shown. (D) Bar plot showing the copy number variation (CNV) of the FOX genes in breast cancers. The PAM50 subtypes are represented by different colors. Bars in the upper panel represent the gain of CNV, and the bottom panel represents the loss of CNV.


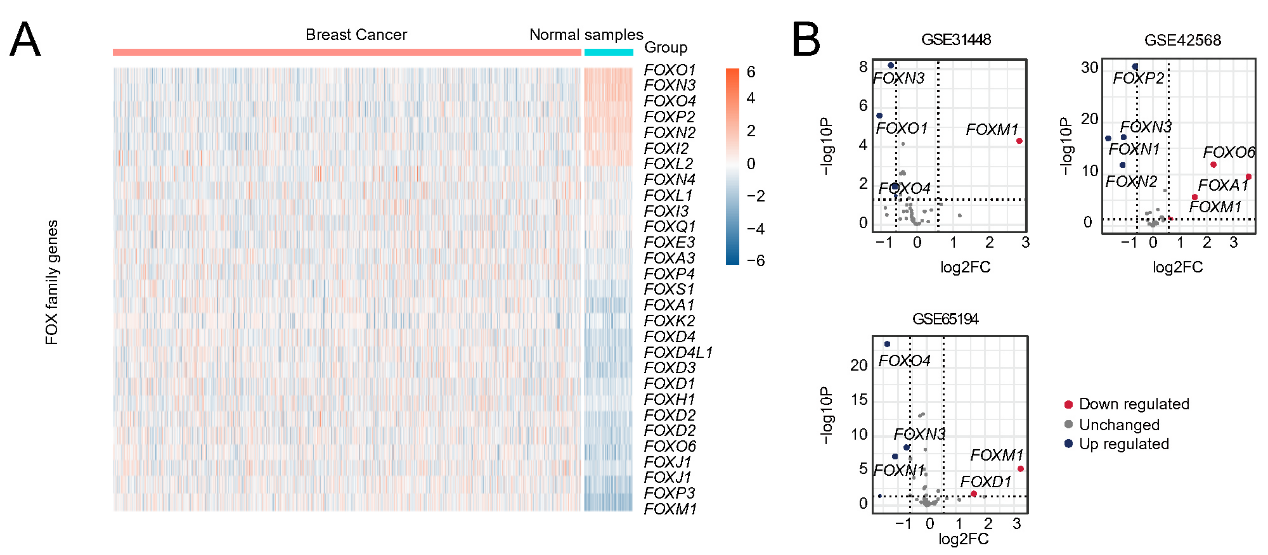


**Figure S2. Transcriptome variation of the FOX gene family in breast cancers.** (A) Heatmap showing the significantly differentially expressed FOX genes between breast cancers and normal samples. (B) Volcano plot illustrating expression of the FOX genes between breast cancers and normal samples in another three independent breast tumor associated datasets.


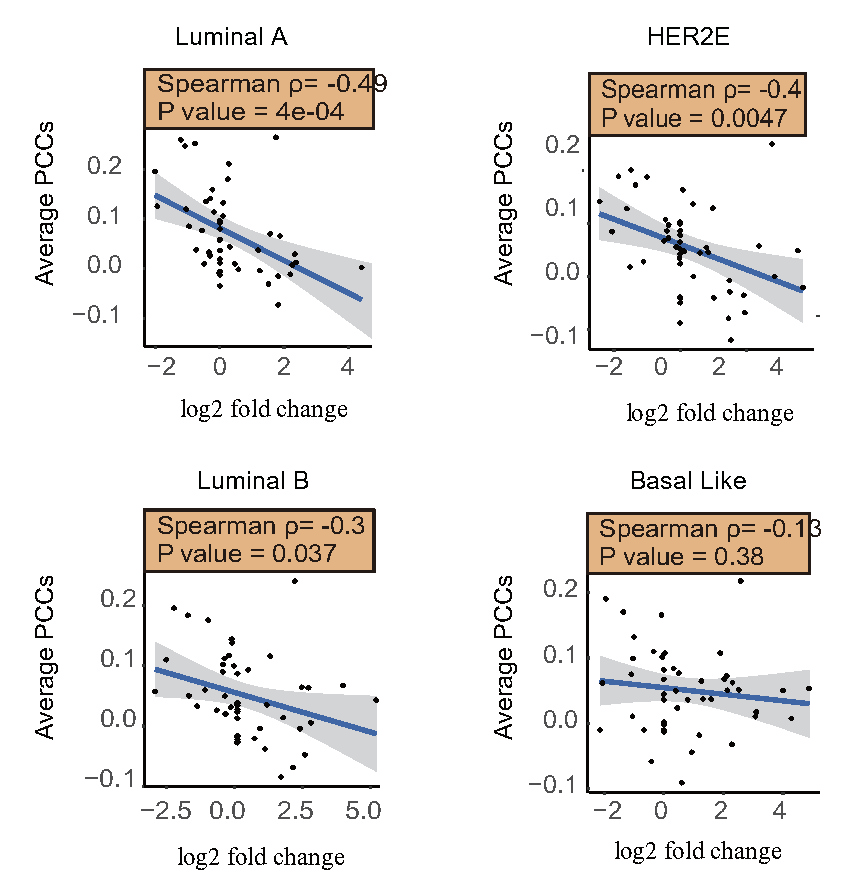


**Figure S3.** Dot plot illustrating a negative relationship between FOX genes and aggregated immune cells.


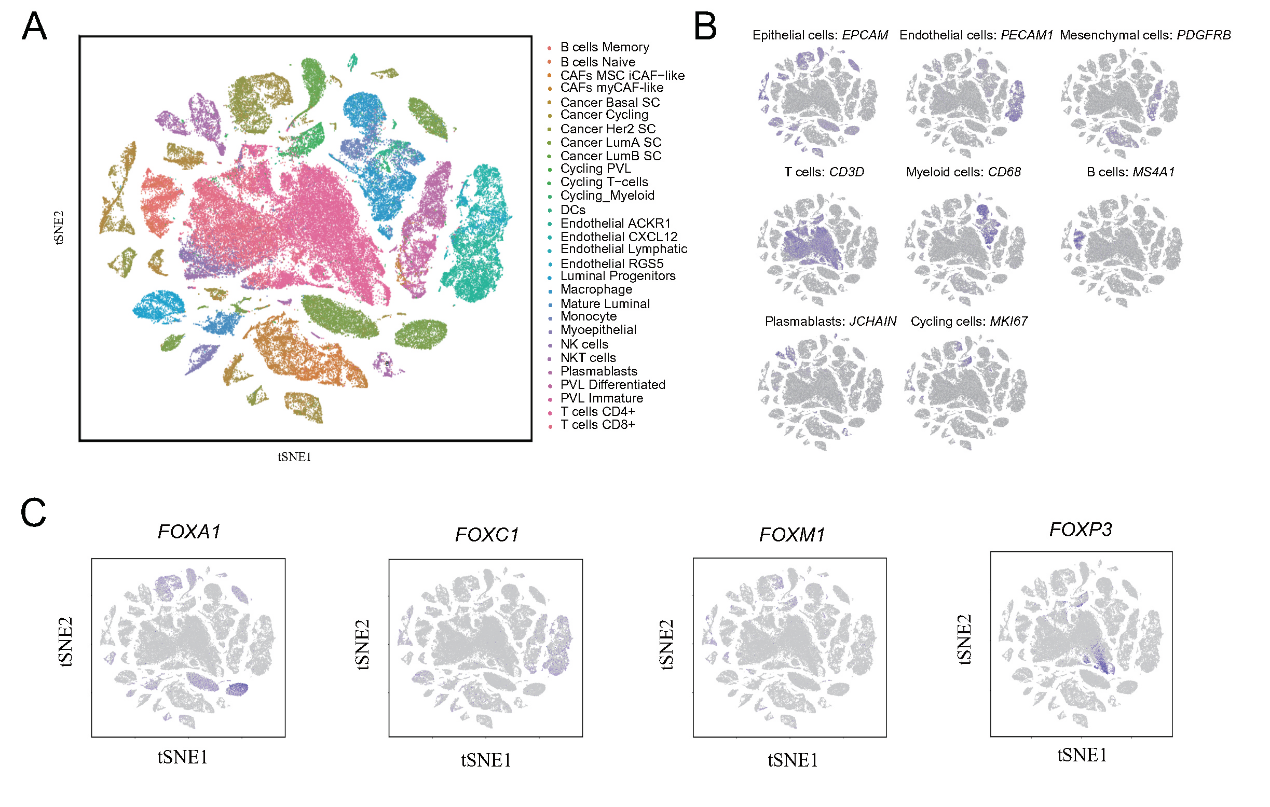


**Figure S4. Cell type specific variation of FOX genes using single-cell RNA sequencing transcriptome.** (A)The t-SNE plot of the major cell types from breast cancer tissues is displayed in the left panel. Cells are colored by cell types. (B). The t-SNE plots of the cell type specific marker genes (B) and essential FOX genes (C) are displayed. Higher gene expression is indicated by deeper blue.

**
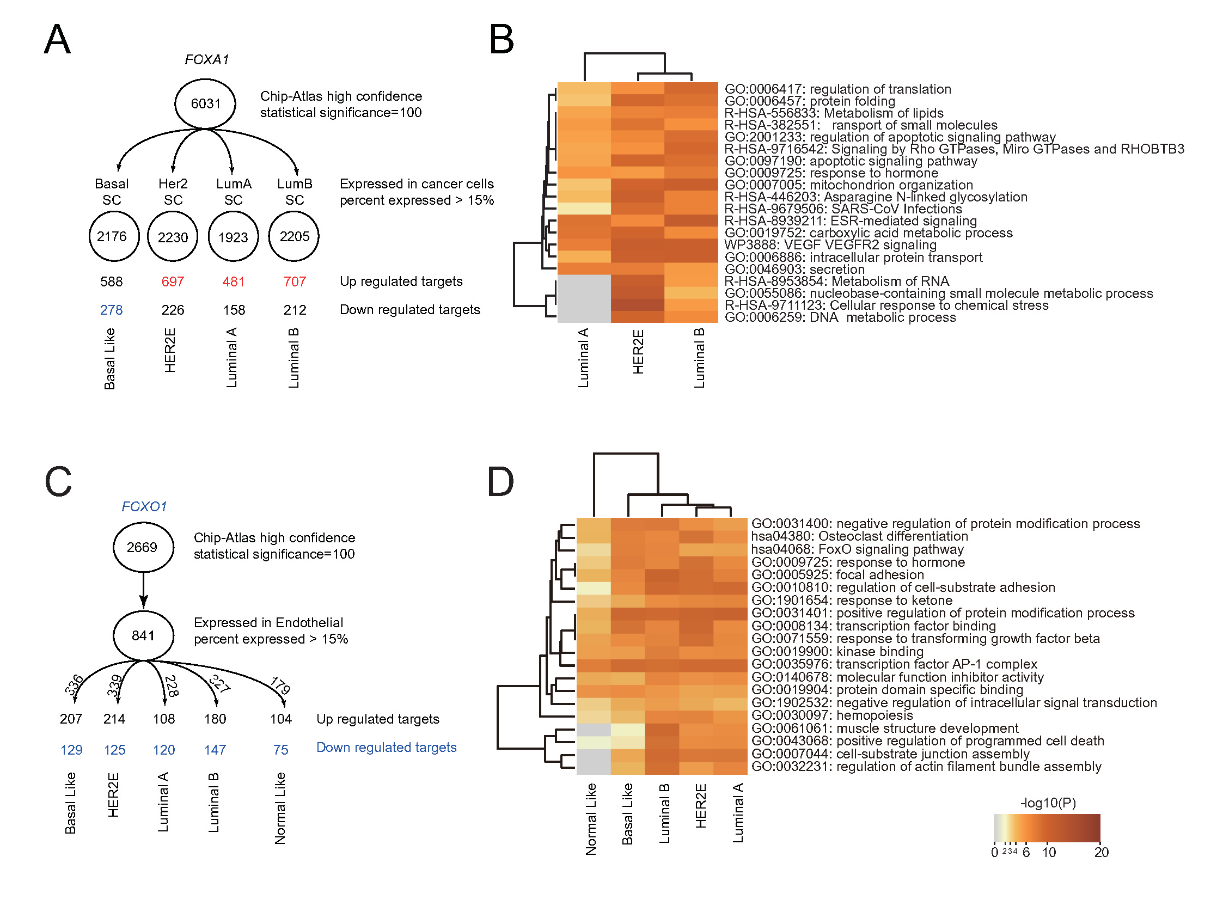
**

**Figure S5. Cell type-specific gene regulation of FOX genes.** (A) The FOXA1 target genes were collected from the Chip-Atlas, and then were filtered according to the percentage of expression in the FOXA1 highly expressed cell types, such as Cancer Basal SC_Cancer epithelial (Basal SC), Cancer LumA SC_Cancer epithelial (LumA SC), Cancer LumB SC_Cancer epithelial (LumB SC), and Cancer Her2 SC_Cancer epithelial (Her2 SC). The significantly differently expressed target genes of FOXA1 in each cell type were defined by their variation between tumor and normal samples. (B) Functional over-representation analysis of the cell type-specific target genes of FOXA1. The similar pipeline was used to define the target genes of FOXO1 (C-D).


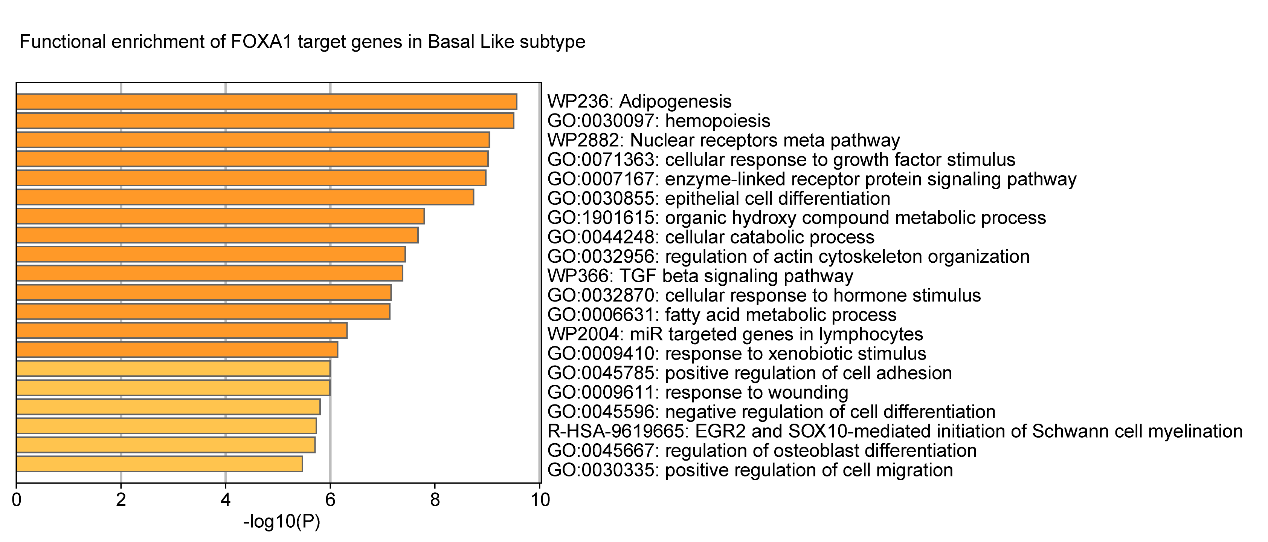


**Figure S6. Functional enrichment of FOXA1 target genes in Basal like subtype.**
